# Heparanase contributes to pancreatic carcinoma progression through insulin-dependent glucose uptake

**DOI:** 10.3389/fcell.2023.1287084

**Published:** 2023-11-22

**Authors:** Alexia Abecassis, Esther Hermano, Adi Yifrach, Aron Popovtzer, Amichay Meirovitz, Michael Elkin

**Affiliations:** ^1^ Sharett Institute of Oncology, Hadassah-Hebrew University Medical Center, Jerusalem, Israel; ^2^ Faculty of Medicine, Hebrew University of Jerusalem, Jerusalem, Israel

**Keywords:** pancreatic cancer, heparanase, diabetes, gemcitabine, glucose uptake

## Abstract

Pancreatic ductal adenocarcinoma (PDAC) is an aggressive tumor, which is highly resistant to existing therapies and characterized by one of the lowest survival rates known for solid cancers. Among the reasons for this poor prognosis are unique pathophysiological features of PDAC, such as dense extracellular matrix [ECM] creating barriers to drug delivery, as well as systemically-deregulated glucose metabolism manifested by diabetic conditions (i.e., hyperinsulinemia/hyperglycemia) occurring in the majority of PDAC patients. Moreover, in addition to systemically deregulated glucose homeostasis, intracellular metabolic pathways in PDAC are rewired toward increased glucose uptake/anabolic metabolism by the tumor cells. While the role of oncogene-driven programs in governing these processes is actively studied, mechanisms linking metabolic dysregulation and ECM enzymatic remodeling to PDAC progression/therapy resistance are less appreciated. The aim of the current study was to investigate the action of heparanase (the predominant mammalian enzyme that degrades heparan sulfate glycosaminoglycan in the ECM), as a molecular link between the diabetic state and the intracellular metabolic rewiring in PDAC pathogenesis. Here we show that in PDAC elevated levels of heparanase, coupled with diabetic conditions typical for PDAC patients, promote growth and chemotherapy resistance of pancreatic carcinoma by favoring insulin receptor signaling and GLUT4-mediated glucose uptake into tumor cells. Collectively, our findings underscore previously unknown mechanism through which heparanase acts at the interface of systemic and intracellular metabolic alterations in PDAC and attest the enzyme as an important and potentially modifiable contributor to the chemo-resistance of pancreatic tumors.

## 1 Introduction

Pancreatic ductal adenocarcinoma (PDAC) is a devastating type of malignancy, with one of the worst prognoses of all solid cancers (Hosein et al., 2022). It develops in the exocrine compartment of the pancreas and accounts for more than 90% of pancreatic cancer cases. In contrast to other tumor types, multiple large-scale trials using targeted agents have been unsuccessful for PDAC, thus chemotherapy (largely, gemcitabine-based regimens) remains the standard of care for patients with advanced PDAC (Amrutkar and Gladhaug, 2017; Lambert et al., 2019; Hosein et al., 2022). Yet, chemotherapy only modestly improves survival, as median OS rates remain at approximately 1 year (Amrutkar and Gladhaug, 2017; Lambert et al., 2019; Hosein et al., 2022). Several mechanisms were proposed to explain PDAC aggressiveness and the limited efficacy of the existing therapies (Raimondi et al., 2009; Lambert et al., 2019). Among them, excessive deposition of desmoplastic extracellular matrix [ECM] posing physical barriers to drug delivery and nutrient/oxygen passage (Hosein et al., 2022), along with altered intracellular metabolism rewired toward increased glucose uptake/anabolic fermentation within the PDAC cells (Ying et al., 2012), attracted the most attention. In parallel with these intratumoral/intracellular phenomena, PDAC is also associated with systemically deregulated glucose homeostasis (i.e., hyperinsulinemia/hyperglycemia, which occurs in a majority of PDAC patients, and frank diabetes in up to 50% of the cases) (Permert et al., 1993; Permert et al., 1997; Stolzenberg-Solomon et al., 2005; Sah et al., 2013; Andersen et al., 2017; Risch, 2019; Deng et al., 2022). Of note, among at least six cancer types attributable to diabetes (Pearson-Stuttard et al., 2018), PDAC and diabetes exhibit a unique bi-directional relationship: epidemiological data point to PDAC as a cause of diabetes; conversely, recent reports suggested that diabetic state sustains the aggressiveness of pancreatic tumors and their resistance to therapies (Stolzenberg-Solomon et al., 2005; Sah et al., 2013; Andersen et al., 2017; Risch, 2019; Deng et al., 2022). Significant progress has been made in deciphering the role of oncogenic signaling and hypoxic/desmoplastic microenvironment in rendering PDAC highly aggressive and resistant to treatment (Ying et al., 2012; Lambert et al., 2019). Less appreciated are mechanisms linking metabolic dysregulation and ECM enzymatic remodeling in PDAC to its progression/therapy resistance.

We have recently demonstrated that a diabetic state induces overexpression of ECM-degrading enzyme heparanase in PDAC (Goldberg et al., 2019). Heparanase is the sole mammalian endoglycosidase capable of hydrolyzing glycosaminoglycan heparan sulfate [HS] in the extracellular space and on the cell surface. Heparanase enzyme is therefore critically involved in the integrity of the ECM, as well as in the modulation of interactions between HS and numerous bioactive molecules (including growth factors and their receptors) at the cell surface (Jayatilleke and Hulett, 2020; Masola et al., 2020; Vlodavsky et al., 2023). In addition, heparanase was shown to stimulate signal transduction pathways, both via its enzymatic, as well as non-enzymatic activities [reviewed in (Vlodavsky et al., 2023)]. Several studies implicated heparanase in pancreatic tumorigenesis, reporting overexpression of the enzyme in PDAC, and its correlation with poor prognosis (Koliopanos et al., 2001; Rohloff et al., 2002; Quiros et al., 2006; Hoffmann et al., 2008). Based on these findings, the inducibility of heparanase expression in PDAC by diabetic milieu components (Goldberg et al., 2019), and impairment of insulin-triggered insulin receptor (INSR) activation under conditions of heparanase deficiency/enzymatic inhibition (Cassinelli et al., 2018; Hermano et al., 2021), we hypothesized that the overexpression of the enzyme can contribute to PDAC pathogenesis by increasing insulin sensitivity of the carcinoma cells, thus mechanistically linking systemic diabetic state to the intracellular metabolic rewiring. Here we report that in the context of hyperinsulinemic conditions [a common phenomenon in PDAC, either in the setting of new onset or long-standing diabetes (Permert et al., 1993; Permert et al., 1997; Stolzenberg-Solomon et al., 2005; Sah et al., 2013)], heparanase augments insulin-INSR signaling cascade in pancreatic tumor cells. We found that elevated levels of heparanase in PDAC tumors when coupled to abundant insulin and glucose content, can empower signal transduction along the insulin-INSR-AKT pathway, promoting PDAC cell proliferation and rendering them resistant to gemcitabine. The underlying molecular events involve augmented glucose uptake by cancer cells due to insulin-dependent translocation of glucose transporter GLUT4 to the plasma membrane, highlighting a previously unknown mechanism through which heparanase enzyme acts at the interface of systemic and intracellular metabolic alterations typical for PDAC.

## 2 Materials and methods

### 2.1 Cell culture

Human pancreatic carcinoma cell lines BXPC3 and PANC1 (well-established PDAC cell lines widely used in pancreatic cancer research, ATCC), authenticated by STR profiling at the Genomics Center of the Biomedical Core Facility, Technion University, Haifa, Israel), and the mouse pancreatic carcinoma cell line Panc02 (Bauer et al., 2007), were grown in RPMI supplemented with 1 mM glutamine, 50 μg/mL streptomycin, 50 U/mL penicillin and 10% FCS (Biological Industries, Beit-Haemek, Israel).

### 2.2 Antibodies

Immunoblot analysis was carried out with the antibodies directed against phospho-INSR Tyr1150/1151 (Cell Signaling 3024L, 1:1000) phospho-AKT Ser 473 (Cell Signaling 4060S, 1:2000), total INSR (Cell Signaling 3020S, 1:1000), total AKT (Cell Signaling 9272S, 1:000), and GAPDH (EnCor 80-010813, 1:20,000). Immunostaining was performed with the antibody directed against GLUT4 (Abcam ab33780, 1:500).

### 2.3 Immunofluorescence

For immunofluorescence analysis, Cy^TM^3 donkey anti-rabbit antibody (The Jackson Laboratory, 1:1000) was used as secondary antibody. Nuclear staining was performed with DAPI (Sigma). Images were captured using a Zeiss LSM 900 confocal microscope and analyzed with Zen software (Carl Zeiss).

### 2.4 Heparanase enzymatic activity assay

Measurements of heparanase enzymatic activity were performed as described previously (Vlodavsky et al., 1983; Lerner et al., 2011). Briefly, equal protein aliquots of cell lysates were incubated with the sulfate-labeled ECM (Vlodavsky et al., 1983; Lerner et al., 2011) for 16 h (37°C, pH 6.2), and the supernatants containing ^35^S -labeled heparan sulfate degradation fragments were analyzed by gel filtration on a Sepharose CL-6B column (0.9 × 30 cm). Fractions (0.2 mL) were eluted with PBS at a flow rate of 5 mL/h and counted for radioactivity. The excluded volume (*V*_*o*_) was marked by blue dextran, and the total included volume (*V*_*t*_) was marked by phenol red. Nearly intact HSPGs are eluted from Sepharose 6B just after the void volume (*K*_av_ < 0.2, fractions 1–10), while HS degradation fragments are eluted toward the *V*_*t*_ of the column (peak II, 0.5 < *K*_av_ < 0.8, fractions 15–35) (Vlodavsky et al., 1983; Lerner et al., 2011). Each experiment was performed at least three times and the variation in elution positions (K_av_ values) did not exceed 15%. Labeled fragments eluted in peak II were shown to be degradation products of HS as they were 5–6-fold smaller than intact HS chains of HSPGs, resistant to further digestion with papain and chondroitinase ABC, and susceptible to deamination by nitrous acid (Vlodavsky et al., 1983). Heparanase activity = *K*_av_ × total cpm in peak II.

### 2.5 Assessment of insulin sensitivity *in vivo*

Ten-week-old male *wt* BALB/c mice (Envigo, Israel) and heparanase-transgenic mice on BALB/c background (Zcharia et al., 2004), were intraperitoneally injected with human insulin (Novo Nordisk, Princeton, NJ, United States; 5  U/kg body weight) or saline (Control) (*n* = 3 per condition). Lysates of skeletal muscle (soleus and gastrocnemius), isolated 10 min after insulin administration, were analyzed by immunoblotting with antibodies directed against phospho-AKT, total AKT, phospho-INSR and total INSR. All experiments were performed in accordance with the Hebrew University Institutional Animal Care and Use Committee.

### 2.6 MTS assay

MTS assay (Promega) was performed according to the manufacturer’s instructions. All treatments were administered to cells cultured in 96-well plates in serum-free DMEM overnight. Each data point shows the mean of pentaplicate cultures.

### 2.7 Glucose uptake assay

The fluorescent analog of glucose 2-deoxy-2-[(7-nitro-2,1,3-benzoxadiazol-4-yl)amino]-D-glucose (2-NBDG; Cayman) was used to measure glucose uptake (Zou et al., 2005). Briefly, PANC1-Vo and PANC1-hpse cells were cultured on 6-well plates for 24 h and then incubated with Choline Chloride Buffer (CCB) without glucose for 2 h. CCB was removed and replaced with fresh CCB containing 100 nM insulin and 50 µM of 2-NBDG. After incubation with 2-NBDG for 45 min, the cells were washed with phosphate-buffered saline (PBS). Subsequently, cells were resuspended by trypsin-EDTA and analyzed using CytoFlex flow cytometer (Beckman Coulter). For the determination of glucose uptake, a minimum of 10,000 cells per sample was collected and analyzed to estimate the percentage of cells that absorbed 2-NBDG. Flow cytometry analysis was performed using CytExpert software.

### 2.8 Statistical analysis

The results are presented as the mean ± SD unless otherwise stated. *p* values ≤ 0.05 were considered statistically significant. Statistical analysis was performed by unpaired Student’s *t*-test, using Microsoft Excel software. Two-sided Fisher test was applied to analyze the relationship between heparanase expression and insulin-induced membrane translocation of GLUT4, using SPSS software (SPSS Inc.).

## 3 Results

**FIGURE 1:**
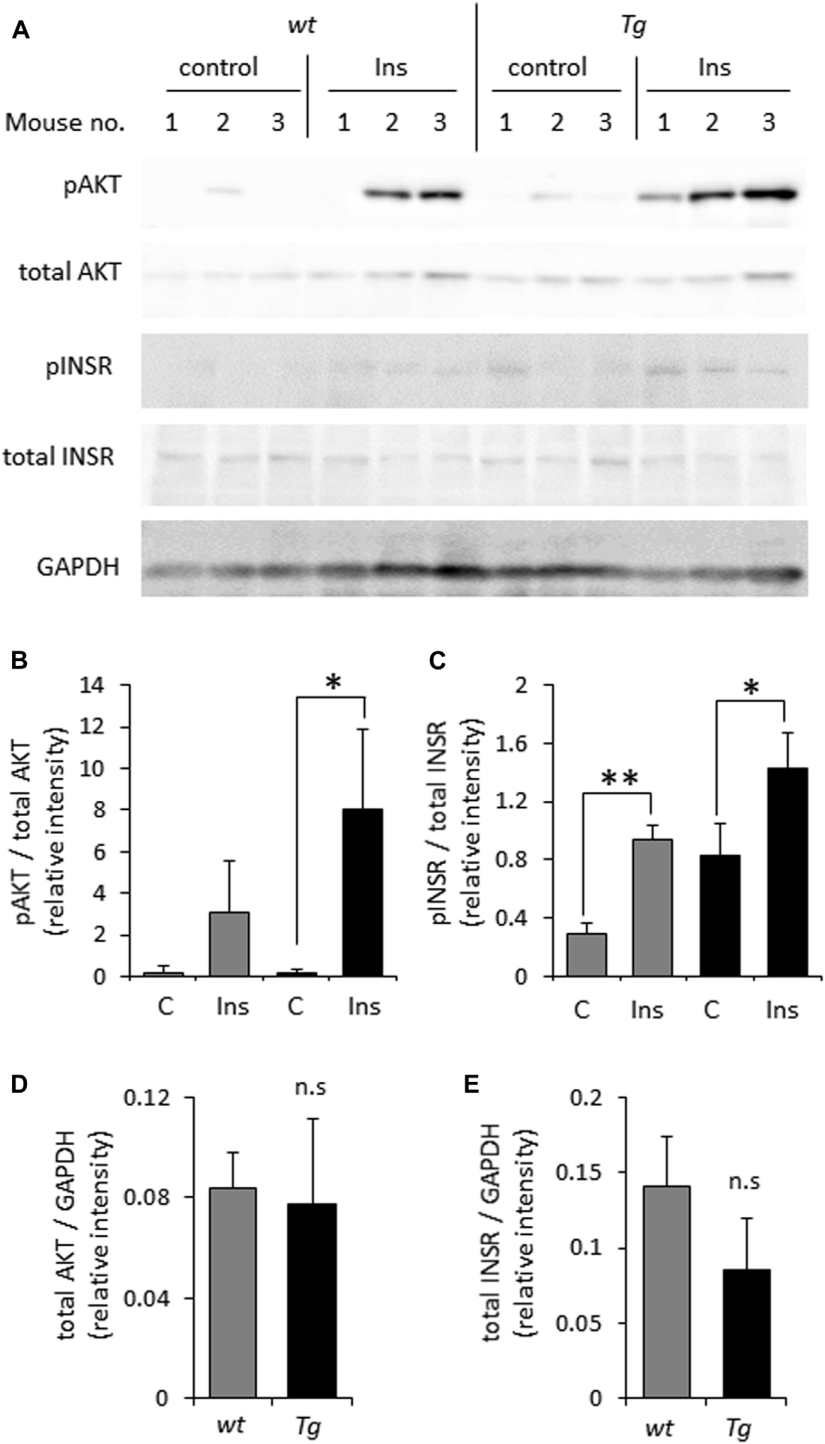
Overexpression of heparanase augments insulin sensitivity *in vivo* **(A)**. Wild-type (*wt*) and heparanase transgenic (Tg) mice were injected (i.p.) with human insulin (Ins) or saline (control, C), as described in Methods. Lysates of skeletal muscle, isolated from the mice 10 min after insulin administration, were analyzed by immunoblotting with antibodies directed against phospho-AKT (pAKT), total AKT, phospho-INSR (pINSR), total INSR and GAPDH **(B–E)**. The band intensity was quantified using ImageJ software. Intensity ratios for pAKT/total AKT **(B)**; pINSR/total INSR **(C)**; total AKT/GAPDH **(D)** and total INSR/GAPDH **(E)** are shown. Data are the mean ± SD. Two-sided Student’s *t*-test *p < 0.05, **p < 0.001; n. s: not statistically significant, *n* = 3 mice per condition.

In light of previously reported impairment of INSR signaling under conditions of heparanase deficiency or enzymatic inhibition (Goldberg et al., 2017; Purushothaman et al., 2017; Cassinelli et al., 2018; Hermano et al., 2021), we first tested whether elevated levels of heparanase enhance INSR signal transduction in prototypic insulin-target tissue (i.e., muscle). For this purpose we utilized soleus skeletal muscle samples derived from heparanase-transgenic mice, overexpressing human heparanase in all tissues (Zcharia et al., 2004), and their *wt* counterparts. As shown in Figure 1, analysis of INSR signaling cascade activation 10 min after i. p. insulin administration in lysates of soleus skeletal muscle samples harvested from heparanase-overexpressing transgenic mice vs. *wt* mice revealed that heparanase overexpression enhanced insulin signaling, as evidenced by increased phospho-INSR [pINSR] and phospho-AKT [pAKT] (Figures 1A–C) levels in soleus skeletal muscle derived from heparanase-transgenic, as compared to *wt* mice. Notably, no difference between protein levels of total INSR and AKT was detected between the muscles of transgenic and *wt* mice prior to insulin administration (Figures 1D, E).

### 3.1 Heparanase enhances INSR signaling and insulin-induced proliferation in pancreatic ductal carcinoma cells

**FIGURE 2:**
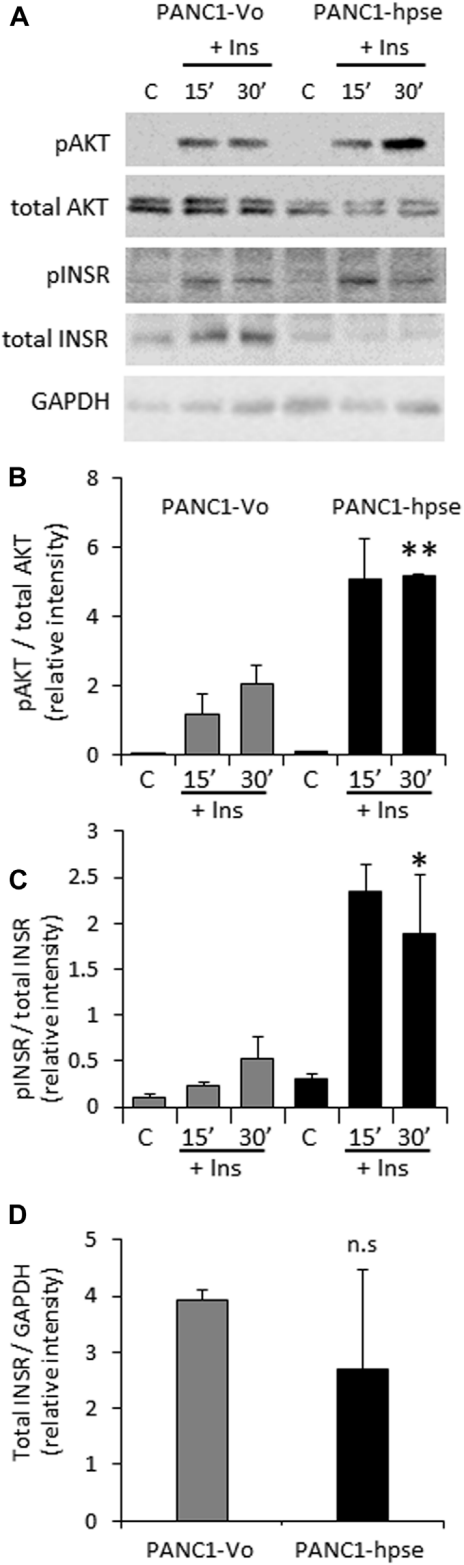
Overexpression of heparanase enhances INSR signaling in human PDAC cells **(A)**. PANC1 cells, stably transfected with vector encoding for human heparanase (PANC1-hpse, black bars) or control empty vector (PANC1-Vo, grey bars) cells were serum-starved overnight and then either remained untreated **(C)** or stimulated with 100 nM insulin (Ins) for 15 and 30 min. Cell lysates containing equivalent amounts of total protein were then immunoblotted using antibody specific for pINSR, pAKT, total INSR, total AKT or GAPDH **(B,C)**. The band intensity was quantified using ImageJ software. Intensity ratios for pAKT/total AKT **(B)**; pINSR/total INSR **(C)** are shown. **(D)** For insulin-untreated PANC1-Vo and PANC1-hpse cells, intensity ratios of total INSR/GAPDH are shown. Data are the mean ± SD. Two-sided Student’s *t*-test *p < 0.05; **p < 0.03; n. s: not statistically significant. The data shown are representative of two **(B)** or three **(C,D)** independent experiments.

Based on the above observations, preferential expression of heparanase in PDAC tumors (Koliopanos et al., 2001; Rohloff et al., 2002; Quiros et al., 2006; Hoffmann et al., 2008), and the presumed role of excessive insulin signaling in pancreatic tumorigenesis (Chan et al., 2014; Zhang et al., 2019; Deng et al., 2022), we hypothesized that the enzyme can also augment INSR signaling in pancreatic carcinoma cells, thus promoting PDAC progression. To interrogate the effect of heparanase overexpression on insulin-INSR pathway in the setting of pancreatic cancer, we first applied PANC1 cells (human PDAC cell line expressing low levels of endogenous heparanase, Supplementary Figure S1) stably transfected with a vector encoding for human heparanase (PANC1-hpse) or control empty vector (PANC1-Vo). Overexpression of heparanase in PANC1-hpse cells was validated by enzymatic activity assay (Supplementary Figure S1). PANC1-hpse and PANC1-Vo cells were either untreated or incubated with insulin, as described in Methods. In agreement with the observations made using muscle (i.e., classical insulin-target tissue, Figure 1), in pancreatic carcinoma cells overexpression of heparanase also resulted in marked augmentation of signaling along insulin-INSR pathway, reflected by increased levels of pINSR and pAKT in PANC1-hpse cells vs. PANC1-Vo cells (Figures 2A–C). Of note, protein levels of total INSR and total AKT in PANC1 cells were not increased by overexpression of heparanase (in fact, the expression levels of total INSR and total AKT were somewhat lower, although not statistically different, in PANC1-hpse, as compared to PANC1-Vo cells, Figure 2).

**FIGURE 3:**
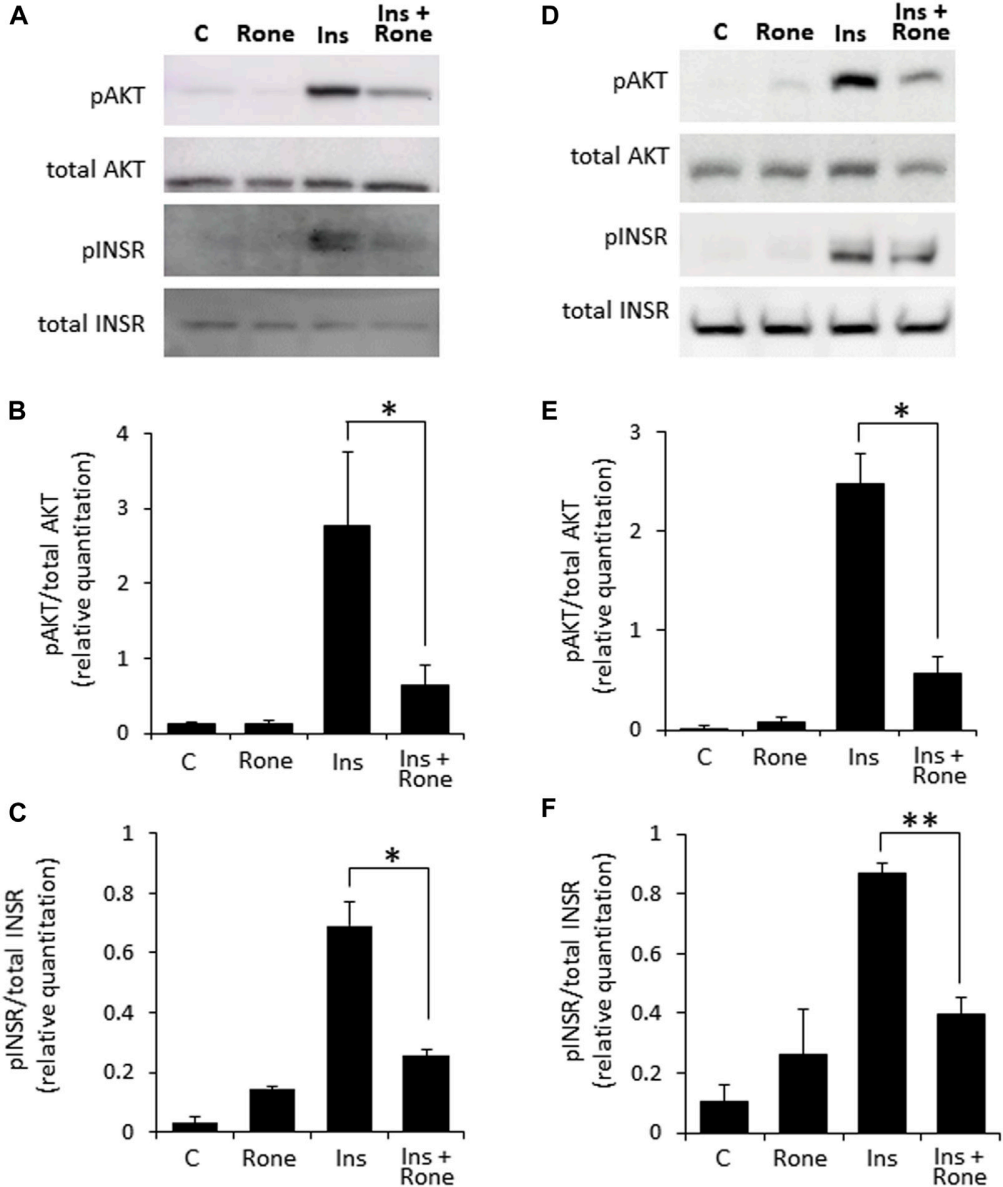
Enzymatic inhibition of heparanase decreases insulin signaling in PDAC cells. PANC1-Hpse **(A–C)** and BxPC3 **(D–F)** human pancreatic carcinoma cells, expressing high levels of heparanase, were serum-starved overnight. Cells were then either remained untreated **(C)** or stimulated with insulin (100 nM, Ins) for 30 min in the absence or presence of specific heparanase inhibitor Roneparstat (100 μg/mL, Rone). Some cells were treated with Roneparstat alone **(A,D)**. Cell lysates containing equivalent amounts of total protein were then immunoblotted using antibody specific for pAKT, total AKT, pINSR or total INSR **(B,C,E,F)**. The band intensity was quantified using ImageJ software. Data are the mean ± SD. Two-sided Student’s *t*-test *p < 0.05; **p < 0.0005; n. s: not statistically significant.

Confirming the involvement of heparanase in enhanced insulin signaling, presence of the specific heparanase enzymatic inhibitor Roneparstat (Noseda and Barbieri, 2020) abolished the effect of heparanase overexpression on insulin signaling (Figures 3A–C). Similarly, Roneparstat inhibited insulin-induced phosphorylation of INSR and AKT in human BXPC3 (Figures 3D–F) and murine Panc02 (Supplementary Figure S2) PDAC cell lines, which express high levels of endogenous heparanase (Supplementary Figure S1).

### 3.2 Heparanase augments tumor-promoting effects of insulin in PDAC cells

**FIGURE 4:**
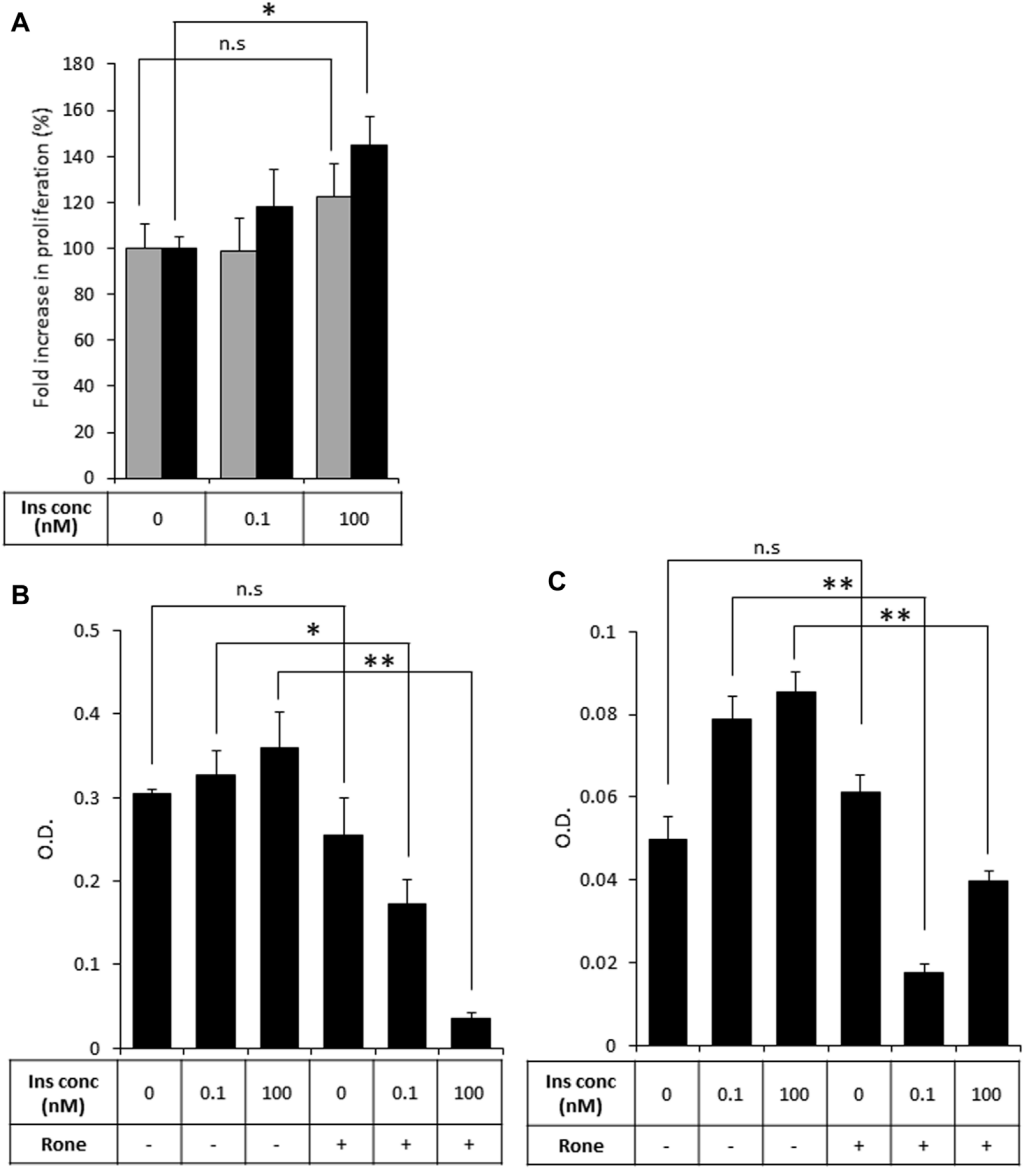
Heparanase promotes mitogenic effect of insulin on PDAC cells **(A)**. PANC1-Vo (grey bars) and PANC1-hpse (black bars) cells were plated on 96-well plates (in pentaplicates) and then cultured for 72 h in the absence or presence of insulin (Ins) at indicated concentrations. Cell growth was analyzed by MTS Cell Proliferation Assay. Note that insulin does not induce statistically significant increase in proliferation of PANC1-Vo cells (lacking heparanase activity). In contrast, in heparanase overexpressing PANC1-Hpse cells proliferation rate was induced by insulin. *p < 0.008 Two-sided Student’s *t*-test. Error bars represent ±SD. The data shown are representative of three independent experiments **(B,C)**. BxPC3 **(B)** and Panc02 **(C)** cells were remained untreated or treated with Roneparstat (200 μg/mL, Rone) and cultured for 72 h in the absence or presence of insulin at indicated concentrations. Cell growth was analyzed by MTS Cell Proliferation Assay. Data are the mean ± SD. Two-sided Student’s *t*-test *p < 0.0002; **p < 0.0005; n. s: not statistically significant.

Then, to test our primary hypothesis that heparanase-mediated augmentation of insulin signaling contributes to PDAC tumor progression, we investigated the biological consequences of insulin stimulation in the presence of increased levels of heparanase in PDAC cells, starting with the mitogenic effect of insulin. Proliferation rate in response to insulin treatment was compared in PANC1-hpse (heparanase-overexpressing) and PANC1-Vo (heparanase-lacking) cells. As shown in Figure 4A (grey bars), treatment with insulin did not result in a statistically significant increase in proliferation of heparanase-lacking PANC1-Vo cells, in agreement with previous reports (Chan et al., 2014; Zhang et al., 2019). However, proliferation of heparanase-overexpressing PANC1-hpse cells was increased in response to insulin (Figure 4A, black bars), when the most pronounced and statistically significant increase was observed at insulin concentrations reflecting hyperinsulinemic conditions frequently present in PDAC patients (Andersen et al., 2017). Conversely, in human (BXPC3) and murine (PANC02) PDAC cell lines, which express high levels of endogenous heparanase (Supplementary Figure S1) heparanase enzymatic inhibitor Roneparstat abolished the mitogenic effect of insulin stimulation (Figures 4B, C).

**FIGURE 5:**
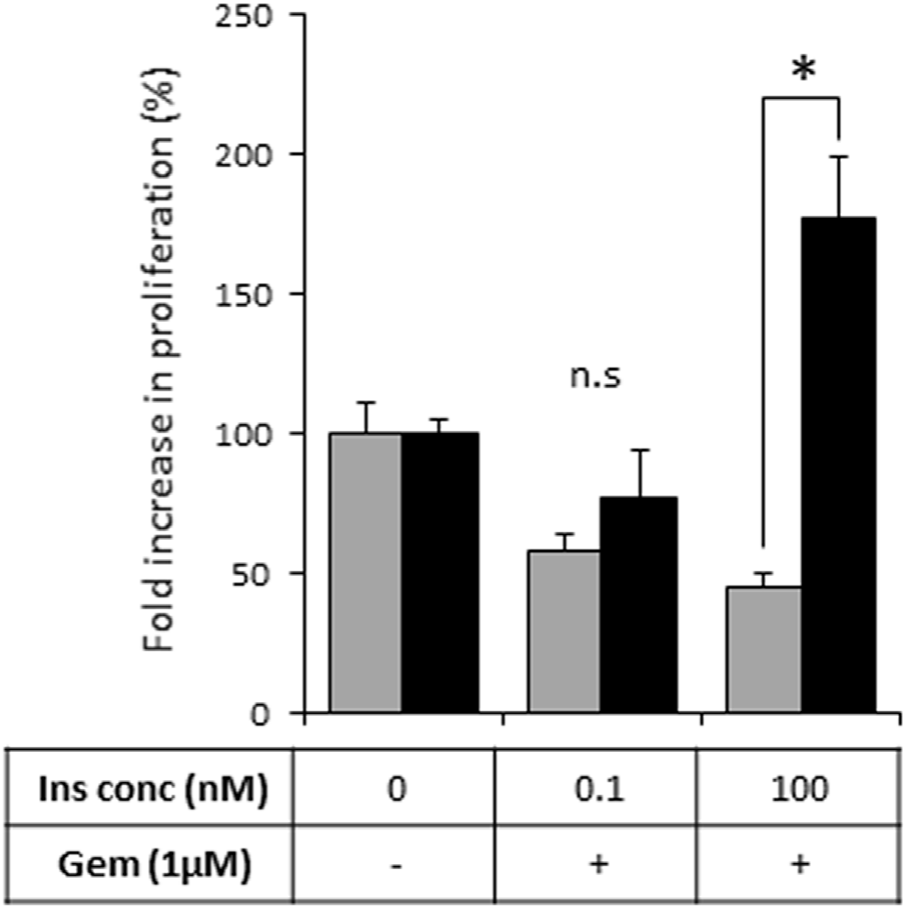
Overexpression of heparanase renders PDAC cells resistant to gemcitabine under hyperinsulinemic conditions. PANC1-Vo (grey bars) and PANC1-hpse (black bars) cells were plated on 96-well plates (in pentaplicates) and cultured either alone or in the presence of Gemcitabine (Gem, 1 microM) for 72 h. To some wells insulin (Ins) was added at concentrations reflecting norminsulinemic and hyperinsulinemic conditions. Cell growth was analyzed by MTS Cell Proliferation Assay. Data are the mean ± SD. Two-sided Student’s *t*-test *p < 0.001; n. s: not statistically significant.

As activation of the insulin-INSR pathway is implicated in the chemoresistance of PDAC tumors (Deng et al., 2022), we next assessed the effects of heparanase-augmented insulin signaling on sensitivity of PDAC cells to gemcitabine—a cornerstone of PDAC treatment at all stages of the disease (Amrutkar and Gladhaug, 2017). As shown in Figure 5, gemcitabine significantly inhibited proliferation of PANC1-Vo cells, and the presence of insulin at various concentrations had no effect on this inhibition. In contrast, in PANC1-hpse cells combination of heparanase overexpression and hyperinsulinemic conditions abolished the inhibitory effect of gemcitabine (Figure 5).

### 3.3 Heparanase overexpression augments insulin-stimulated glucose uptake by PANC1 cells

**FIGURE 6:**
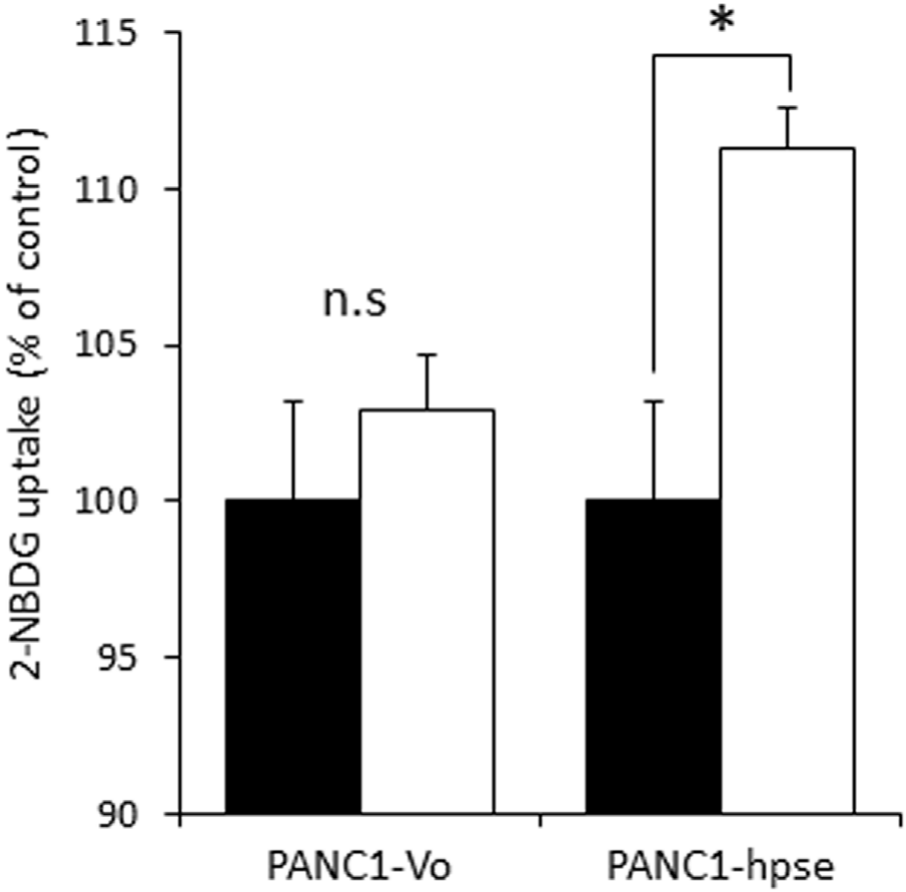
Heparanase promotes insulin-stimulated glucose uptake in PDAC cells. Uptake of glucose analog 2-NBDG in untreated (black bars) and insulin stimulated (white bars) PANC1-Vo and PANC-Hpse cells was determined as described in Methods. Data are the mean ± SD. Two-sided Student’s *t*-test *p < 0.01; n. s: not statistically significant.

Although several molecular mechanisms of gemcitabine resistance, encompassing different pathways, have been suggested (Deng et al., 2022), recent studies highlighted the role of metabolic alterations in PDAC cells in conferring gemcitabine resistance. These alterations depend on augmented glucose uptake, which enables increased glycolysis to generate the biosynthetic intermediates for *de novo* synthesis of pyrimidine nucleotides, which in turn render gemcitabine ineffective by molecular competition (Shukla et al., 2017). We therefore assumed that heparanase-mediated enhancement of insulin signaling leads to increased glucose uptake by PDAC cells to convey resistance to gemcitabine. To test this assumption we compared insulin-stimulated glucose uptake in PANC1-hpse and PANC1-Vo cells using fluorescent 2-NBDG glucose analog. As shown in Figure 6, overexpression of heparanase augmented insulin-stimulated glucose uptake in PANC1-Hpse, as compared to PANC1-Vo cells.

**FIGURE 7:**
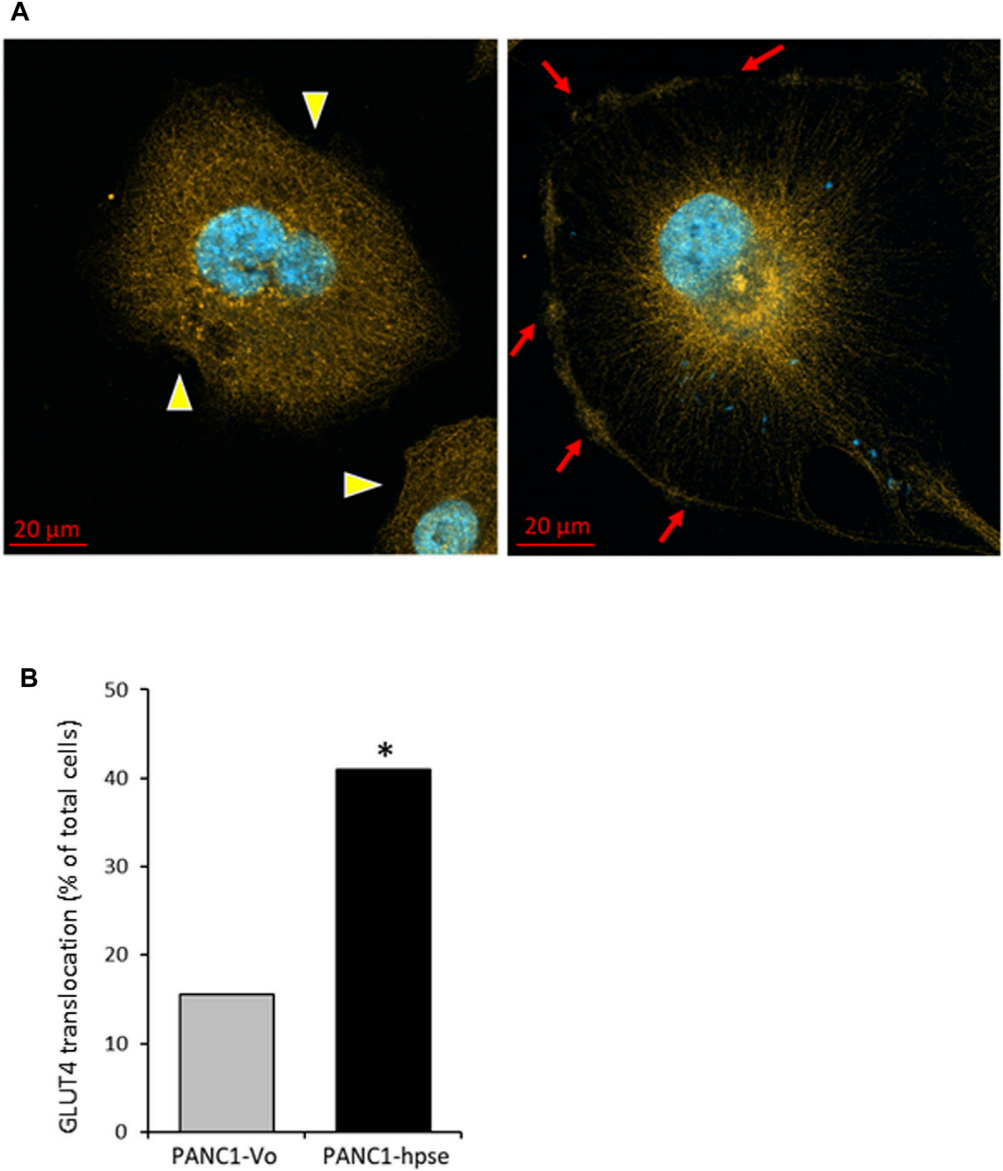
Insulin stimulation results in augmented translocation of GLUT4 to the plasma membrane in heparanase overexpressing pancreatic carcinoma cells **(A)**. Distribution of GLUT4 in PANC1-Vo (left) and PANC-Hpse (right) cells, 45 min after insulin stimulation (100 nM) were investigated by confocal microscopy. Note occurrence of translocation of GLUT4 protein to the plasma membrane (red arrows) following insulin stimulation in PANC1-Hpse cells, as compared to the PANC-Vo cells, in which GLUT4 remained at perinuclear location (yellow arrowheads) **(B)**. PANC1-Vo (grey bars) and PANC1-Hpse (black bars) cells with membrane-translocated GLUT4 were quantified in ≥4 microscopic fields per slide, at least 3 slides per condition, and the percentage of cells with membrane-translocated GLUT4 was calculated. Note significantly higher percentage of membrane-translocated GLUT4 in PANC1-Hpse cells, as compared to the PANC-Vo cells; two sided Fisher test *p < 0.005.

As stated above, the insulin-induced uptake of glucose into cells is mediated by the glucose transporter family (GLUTs) (Ancey et al., 2018). Insulin can induce both long-term and acute glucose uptake into cells (Waldhart et al., 2017; Hoxhaj and Manning, 2020). Among the known GLUTs, insulin-independent GLUT1 is widely distributed in different tissues, and often elevated in many cancer types (Hoxhaj and Manning, 2020). The main insulin-dependent transporter GLUT4 is responsible for the majority of glucose transport into muscle and adipose cells. In the absence of insulin, GLUT4 exists predominately perinuclearly, but insulin-triggered AKT activation mediates rapid GLUT4 translocation to the cell membrane for active transport of glucose into the cells, to function in plasma glucose clearance after food intake [reviewed in (Hoxhaj and Manning, 2020)]. Interestingly, GLUT4 is expressed at relatively high levels in PDAC tumors (Cheng et al., 2018) and pancreatic carcinoma cell lines (Dey et al., 2012; Krapf et al., 2020). Since in our experiments heparanase-enhanced insulin signaling induced rapid glucose uptake, we next compared membrane translocation of GLUT4 in response to insulin in PANC1-hpse vs. PANC1-Vo cells. As shown in Figure 7, confocal microscopy analysis of GLUT4 cellular localization under basal condition vs. insulin stimulation revealed markedly increased translocation of GLUT4-to the membrane upon insulin stimulation in heparanase-overexpressing PANC1-Hpse cells, as compared with the PANC1-Vo cells (Figure 7). Taken together, these findings suggest that in PDAC co-occurrence of heparanase upregulation [i.e., due to induction by diabetic milieu components (Goldberg et al., 2019)], abundance of insulin and expression of GLUT4 create optimal conditions for triggering enhanced signaling along INSR-AKT-GLUT4 axis that promote PDAC progression/therapy resistance (among other mechanisms) through increased uptake of glucose.

## 4 Discussion

Augmented glucose influx is an essential prerequisite for its increased fermentation in cancer cells via the glycolytic pathway (i.e., Warburg effect), well-documented in PDAC, as well as other types of malignancies (Ying et al., 2012; Shukla et al., 2017; Ancey et al., 2018; Hoxhaj and Manning, 2020). Notably, in PDAC patients this metabolic shift often occurs in the face of systemic deterioration in glycemic control manifested by hyperinsulinemia/hyperglycemia (Permert et al., 1993; Permert et al., 1997; Stolzenberg-Solomon et al., 2005; Sah et al., 2013; Andersen et al., 2017; Risch, 2019; Deng et al., 2022). Furthermore, while several types of malignancies have been associated with impaired glucose metabolism (Pearson-Stuttard et al., 2018), relationships between PDAC and diabetes are unique due to the postulated reverse causality between these two devastating diseases. Indeed, PDAC is considered a cause of diabetes in a significant fraction of patients; in turn, diabetic conditions reportedly promote pancreatic tumorigenesis and resistance to treatment (Stolzenberg-Solomon et al., 2005; Sah et al., 2013; Andersen et al., 2017; Risch, 2019; Deng et al., 2022). Nevertheless, the mechanistic data concerning relationships between PDAC progression and systemic deterioration of glycemic control, as well as the influence of these relationships on metabolic alterations within PDAC cells are rather scarce. Moreover, the contribution of ECM-degrading enzymes to these relationships was not previously investigated. Here we show that in PDAC elevated levels of heparanase [the predominant mammalian enzyme that degrades HS glycosaminoglycan at the ECM/cell surface (Jayatilleke and Hulett, 2020)], coupled to diabetic conditions typical for PDAC (Stolzenberg-Solomon et al., 2005; Dey et al., 2012; Sah et al., 2013; Andersen et al., 2017; Cheng et al., 2018; Krapf et al., 2020; Deng et al., 2022), promote growth and therapy resistance of pancreatic carcinoma cells by fostering INSR signaling and GLUT4 mediated glucose uptake.

In fact, the involvement of heparanase in diabetes and its complications was actively investigated during the last decade (Baker et al., 2010; Gil et al., 2012; Goldberg et al., 2014; Abu El-Asrar et al., 2015; Simeonovic et al., 2020). Previous findings demonstrated essential role of the enzyme in ensuring insulin sensitivity in the insulin-target tissues [i.e., muscle, (Hermano et al., 2021)]. A number of studies also linked heparanase activity to the augmented INSR signaling in several types of malignant tumors (i.e., myeloma, breast carcinoma, synovial sarcoma) (Goldberg et al., 2017; Purushothaman et al., 2017; Cassinelli et al., 2018). Important to note that under certain experimental conditions heparanase can also exert non-enzymatic effects, playing a part in the activation of selected signaling molecules, including AKT, Src, and EGFR (Vlodavsky et al., 2023). Of those the best documented is the induction of AKT phosphorylation [reviewed in (Vlodavsky et al., 2023)]. Since AKT is a key downstream mediator of insulin-INSR signal transduction and GLUT4 translocation, it might be anticipated that augmentation of insulin signaling by heparanase is influenced by its non-enzymatic action as well. Interestingly, the previous studies focusing on heparanase-facilitated insulin responses in various experimental systems reported that enzymatic, rather than non-enzymatic action of heparanase is responsible for this phenomenon, as enzymatically-inactive forms of heparanase had no effect on signal transduction events along insulin/INSR axis, and enzymatic inhibitors of heparanase diminished heparanase-facilitated response to insulin (Goldberg et al., 2017; Purushothaman et al., 2017; Cassinelli et al., 2018). Noteworthy, we were unable to detect changes in phospho-AKT levels in heparanase-overexpressing tissue (Figure 1B) and cell (Figure 2B) lysates in the absence of insulin stimulation. Nevertheless, given the previously documented role of the signaling molecules (such as Src, EGFR) in metabolic plasticity of the cancer cell [i.e., through regulation of master glycolytic transcription factors, expression of insulin-independent glucose transporters, modulation of glycolytic enzyme activity (Sigismund et al., 2018; Pelaz and Tabernero, 2022)], along with the ability of heparanase to affect activation status of these signaling molecules in enzymatic-independent fashion, the contribution of non-enzymatic effects of heparanase to the metabolic alterations occurring in PDAC tumorigenesis could not be excluded.

As stated above, connection between PDAC and diabetes is distinctive in its reciprocality, contrasting other diabetes-influenced cancer types (Pearson-Stuttard et al., 2018). While the diabetes-promoting action of PDAC is explained by several studies as a paraneoplastic phenomenon caused by specific PDAC-secreted mediators (Sah et al., 2013; Batabyal et al., 2014; Javeed et al., 2015; Sagar et al., 2016; Andersen et al., 2017), our present findings provide an explanation for the PDAC-promoting action of diabetes, emphasizing the role of heparanase. Indeed, we documented an increase in signal transduction response to insulin along the INSR-AKT axis in heparanase-overexpressing muscle tissue (prototypic insulin target organ, Figure 1), and in a similar manner–in heparanase-overexpressing PDAC cells (Figure 2). This increase, and the resulting proliferative response of PDAC cells (Figure 4) was attenuated in the presence of specific inhibitor of the enzyme (Figures 3, 4B, C). Importantly, we detected that heparanase-driven increase in insulin-triggered signaling events within PDAC cells rendered them resistant to gemcitabine treatment (Figure 5) and revealed that underlying molecular events involve enhanced intracellular uptake of glucose (Figure 6) due to membrane translocation of insulin-dependent GLUT4 transporter (Figure 7). When taken together with the previous observations that heparanase overexpression in PDAC is induced by diabetic milieu components (Goldberg et al., 2019), these findings provide new insight into the role of the enzyme in shaping bi-directional connection between PDAC progression and intratumoral/systemic metabolic changes. Besides induction of heparanase in pancreatic tumor cells due to PDAC-triggered impairment of glucose homeostasis (Goldberg et al., 2019), this connection involves heparanase-empowered activation of the INSR-AKT signaling cascade. Together with an additional defining element of PDAC, i.e., increased insulin levels, this activation allows augmented uptake of glucose by the tumor cells through GLUT4 membrane translocation, thus promoting growth and chemoresistance of PDAC. However, the limitation of our study is that the precise mode of the enzyme action in fostering INSR signaling remained largely unexplained. The ability of heparin/HS (including heparanase-generated soluble HS fragments) to promote/stabilize growth factor tyrosine kinase receptor-ligand binding, dimerization and signal transduction (Spivak-Kroizman et al., 1994; Vlodavsky et al., 1996; Elkin et al., 2001; Derksen et al., 2002; Kemp et al., 2006; Lemmon and Schlessinger, 2010) provides one conceivable explanation, suggesting that in the similar manner, HS degradation fragments released by the enzyme facilitate insulin-INSR signaling complexes. Of note, the key extracellular HS proteoglycan perlecan was reported to modulate insulin responses, as loss of perlecan results in augmented insulin sensitivity in muscle (Yamashita et al., 2018). Given the ability of heparanase to reduce the amount of extracellular HS (Elkin et al., 2001; Hermano et al., 2014), perlecan degradation by the enzyme can explain increased insulin sensitivity in heparanase-overexpressing PDAC cells. Additionally, cell-surface HS proteoglycan glypican-4 reportedly enhances insulin signaling via interaction with the INSR (Ussar et al., 2014). As enzymatically-regulated release of glypican from the cell surface appears to be required to enable its direct interaction with INSR (Ussar et al., 2014), it is plausible that heparanase [known to drive shedding of cell surface HS proteoglycans (Ramani et al., 2013)] can facilitate this process. Clearly, further studies are warranted to fully characterize molecular events underlying heparanase-augmented insulin signaling. An additional limitation of the present study is that no *in vivo* model was used to validate the effect of heparanase-augmented insulin signaling on the chemo-resistance of PDAC cells to gemcitabine. This is due to the lack of immunodeficient mouse host models faithfully reflecting concurrent chronic hyperinsulinemia and PDAC progression. Yet, our present findings attest heparanase enzyme as an important and potentially modifiable contributor to progression/chemo-resistance of PDAC, acting along INSR-AKT-GLUT4 axis. Moreover, as the recent attempts to translate direct targeting of AKT pathway into clinic were proven to be highly challenging (He et al., 2021), heparanase-inhibiting approaches [which are currently under active development (Rivara et al., 2016; He et al., 2022)] may offer an appealing tactic to sensitize pancreatic tumors to gemcitabine, as well as uncouple PDAC and diabetes cancer progression in a significant fraction of patients.
