# Supplementary material for: Heparanase contributes to pancreatic carcinoma progression through insulin-dependent glucose uptake
Source: Front Cell Dev Biol. 2023 Nov 22;11:1287084. doi: 10.3389/fcell.2023.1287084 (PMC10702555; doi:10.3389/fcell.2023.1287084)
Supplement: Supplementary file 1 [file DataSheet2.PDF]

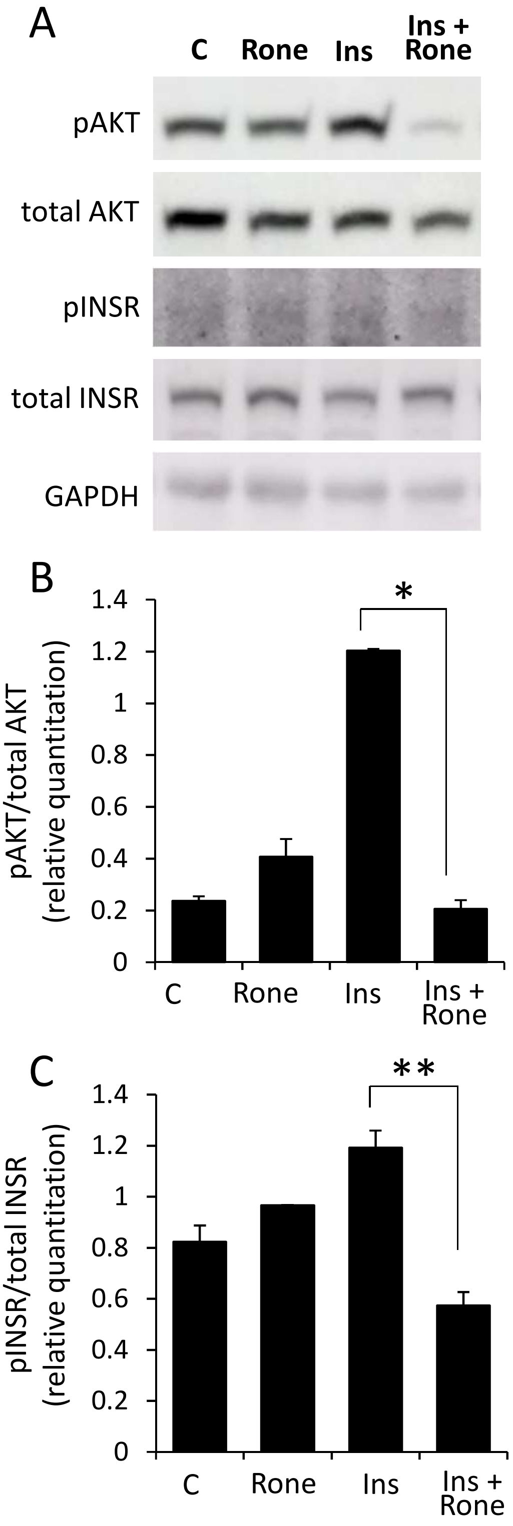

Supplementary Figure 2. Enzymatic inhibition of heparanase decreases insulin signaling in Panc02 murine pancreatic carcinoma cells. Panc02 cells, expressing high levels of endogenous heparanase, were serum-starved overnight and then either remained untreated (**C**) or stimulated with insulin (100 nM, Ins) for 30 minutes in the absence or presence of specific heparanase inhibitor Roneparstat (100 µg/ml, Rone). Some cells were treated with Roneparstat alone. **A.** Cell lysates containing equivalent amounts of total protein were then immunoblotted using antibody specific for pAKT, total AKT, pINSR, total INSR, or GAPDH. **B, C,** The band intensity was quantified using ImageJ software. Data are the mean±SD. Two-sided Student's t test *p<0.002; **p<0.02; n.s: not statistically significant.
