# Supplementary material for: Heparanase contributes to pancreatic carcinoma progression through insulin-dependent glucose uptake
Source: Front Cell Dev Biol. 2023 Nov 22;11:1287084. doi: 10.3389/fcell.2023.1287084 (PMC10702555; doi:10.3389/fcell.2023.1287084)
Supplement: Supplementary file 2 [file DataSheet1.PDF]

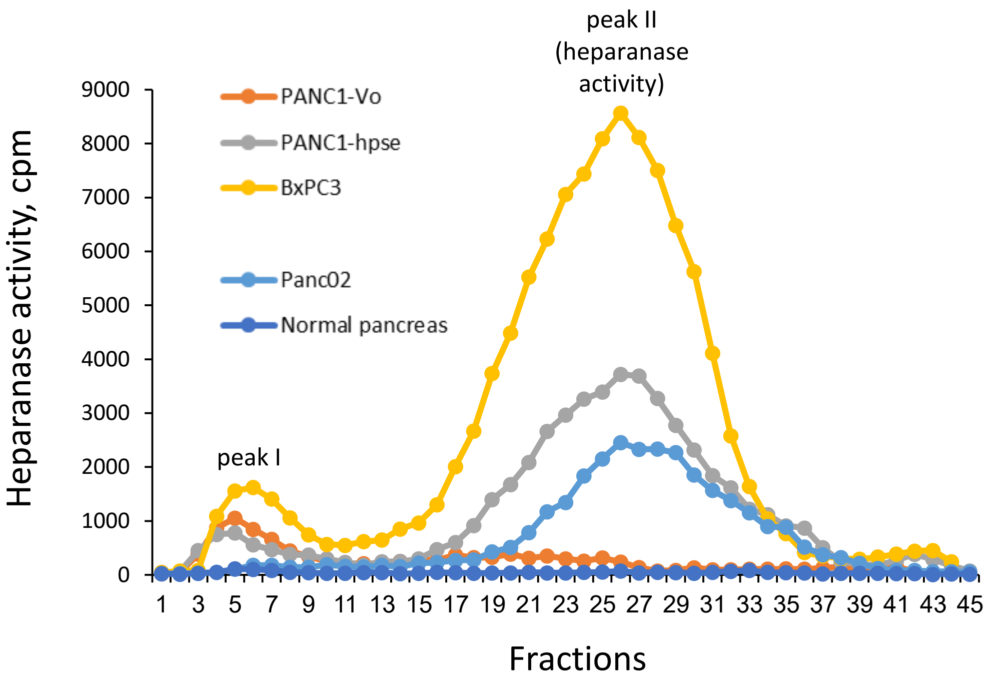

Supplementary figure 1. Heparanase enzymatic activity in human and murine PDAC cells. Equal protein aliquots of human PDAC cell lines BxPC3 and Panc1 **[**stably transfected with vector encoding for human heparanase under constitutive CMV promoter (PANC1-hpse) or empty vector (PANC1-Vo)], mouse PDAC line Panc02, as well as normal mouse pancreas, were incubated in dishes coated with ^{35}S-labeled ECM, prepared as described in Methods. Sulfate-labeled material released into the incubation medium was analyzed by gel filtration on a Sepharose 6B column and quantitation of heparanase activity was performed as described in Methods.

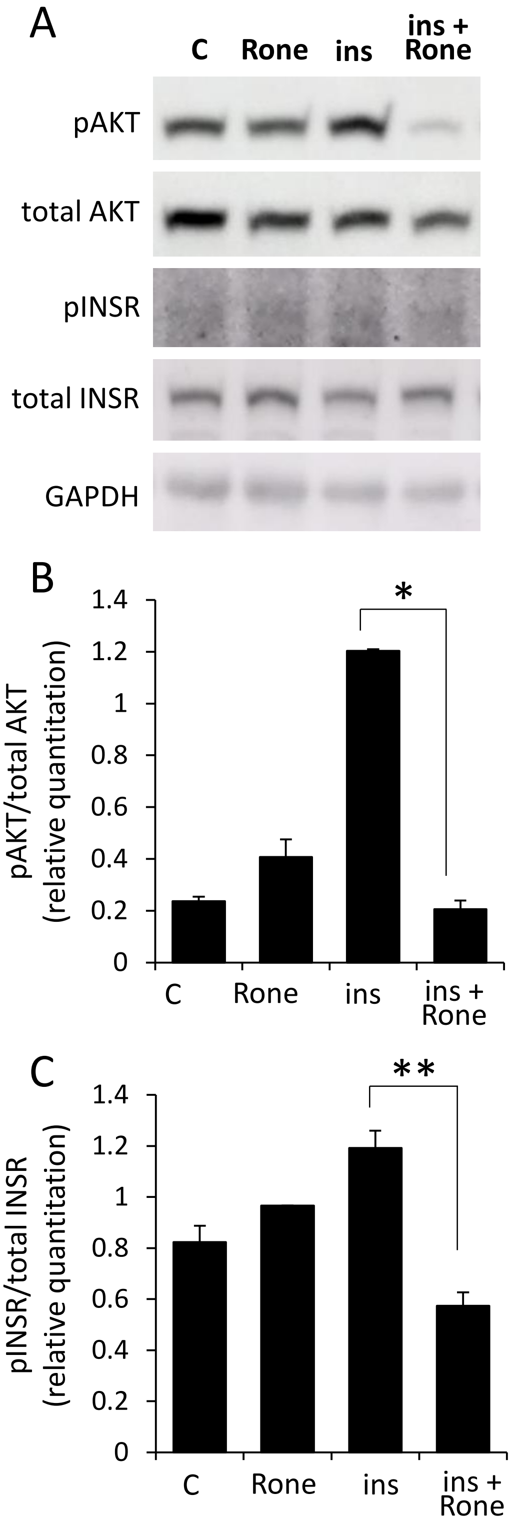

Supplementary Figure 2. Enzymatic inhibition of heparanase decreases insulin signaling in Panc02 murine pancreatic carcinoma cells. Panc02 cells, expressing high levels of endogenous heparanase, were serum-starved overnight and then either remained untreated (**C**) or stimulated with insulin (100 nM, **ins**) for 30 minutes in the absence or presence of specific heparanase inhibitor Roneparstat (100 μg/ml, **Rone**). Some cells were treated with Roneparstat alone. **A.** Cell lysates containing equivalent amounts of total protein were then immunoblotted using antibody specific for pAKT, total AKT, pINSR, total INSR, or GAPDH. **B, C,** The band intensity was quantified using ImageJ software. Data are the mean±SD. Two-sided Student's t test *p<0.002; **p<0.02; n.s: not statistically significant.
